# Alcohol-Induced Psychosis: Beyond Korsakoff. Case report and Literature Review

**DOI:** 10.1192/j.eurpsy.2024.1002

**Published:** 2024-08-27

**Authors:** Á. De Vicente Blanco, B. Orgaz Álvarez, P. Ibáñez Mendoza, M. Velasco Santos, J. Garde González

**Affiliations:** Hospital Universitario La Paz, Madrid, Spain

## Abstract

**Introduction:**

Chronic consumption of alcohol has clear deleterious effects on the nervous system. Among its less-recognized consequences are subacute and chronic alcohol-induced psychotic disorders. Lasègue, Garnier, Magnan, and Michaux provided exhaustive clinical descriptions of different presentations of *subacute alcoholic delusional disorder*, while Kraepelin, Allamagny, and Neveu defined the characteristics of *chronic alcoholic hallucinatory psychosis*. Both conditions are characterized by the occurrence of hallucinations and vivid dream-like content in their delusions, along with potential emotional detachment from the symptoms. Presently, both conditions are categorized under the generic term ‘Alcohol-Induced Psychotic Disorder,’ with limited available scientific literature.

**Objectives:**

Our goal is to bring attention to the existence of subacute and chronic alcohol-induced psychosis in individuals with long-term alcohol users.

**Methods:**

Case report using clinical records and a non-systematic literature review.

**Results:**

A 63-year-old male, with a forty-year history of chronic alcoholism and no other prior mental health issues, was admitted in the emergency department. He conveyed vague delusional notions regarding his roommate and described vivid morning dreams in which he tried to communicate but couldn’t speak. This led him to believe his roommate harboured harmful intentions. Additionally, he mentioned that for the past two months, he had developed a telepathic connection with his sister and his deceased mother, with whom he felt he communicated without speaking. He described feeling strangement and anxiety concerning these experiences, which he firmly believed to be undeniably real. He reported being able to hear the voices of his mother and sister. He also described short-term memory problems dating back two years. He denied any other psychopathology and exhibited probable ideational and emotional impoverishment secondary to chronic alcohol consumption. Confirmation of the patient’s account was provided by his family members. The prescribed treatment included antipsychotic medication and a recommendation for alcohol abstinence.

**Conclusions:**

Descriptions of chronic and subacute alcohol-induced psychoses are found in early psychiatric textbooks but have been omitted from contemporary classifications. While their incidence is low among chronic alcohol users, they represent a severe clinical entity. These disorders are usually distinguished by the presence of delusions and vivid hallucinations characterized by dream-like content. This distinct symptomatology aids in the accurate differentiation from other psychotic disorders and clinicians should be aware of their existence.

**Disclosure of Interest:**

None Declared

